# Disclosure control of machine learning models from trusted research environments (TRE): New challenges and opportunities

**DOI:** 10.1016/j.heliyon.2023.e15143

**Published:** 2023-04-03

**Authors:** Esma Mansouri-Benssassi, Simon Rogers, Smarti Reel, Maeve Malone, Jim Smith, Felix Ritchie, Emily Jefferson

**Affiliations:** a University of Dundee, United Kingdom; b NHS National Services Scotland, United Kingdom; c University of the West of England, United Kingdom; d Health Data Research (HDR), United Kingdom

**Keywords:** Trusted research environment, Safe haven, AI, Machine learning, Data privacy, Disclosure control

## Abstract

**Introduction:**

Artificial intelligence (AI) applications in healthcare and medicine have increased in recent years. To enable access to personal data, Trusted Research Environments (TREs) (otherwise known as Safe Havens) provide safe and secure environments in which researchers can access sensitive personal data and develop AI (in particular machine learning (ML)) models. However, currently few TREs support the training of ML models in part due to a gap in the practical decision-making guidance for TREs in handling model disclosure. Specifically, the training of ML models creates a need to disclose new types of outputs from TREs. Although TREs have clear policies for the disclosure of statistical outputs, the extent to which trained models can leak personal training data once released is not well understood.

**Background:**

We review, for a general audience, different types of ML models and their applicability within healthcare. We explain the outputs from training a ML model and how trained ML models can be vulnerable to external attacks to discover personal data encoded within the model.

**Risks:**

We present the challenges for disclosure control of trained ML models in the context of training and exporting models from TREs. We provide insights and analyse methods that could be introduced within TREs to mitigate the risk of privacy breaches when disclosing trained models.

**Discussion:**

Although specific guidelines and policies exist for statistical disclosure controls in TREs, they do not satisfactorily address these new types of output requests; i.e., trained ML models. There is significant potential for new interdisciplinary research opportunities in developing and adapting policies and tools for safely disclosing ML outputs from TREs.

## 1 Introduction

Trusted Research Environments (TRE)s (also termed Safe Havens) are secure and safe platforms that enable researchers to access and analyse personal data [1]. An example is the network of Scottish research Safe Havens, in which researchers can access a wide range of health records for Scottish individuals [2]. TREs follow a portfolio approach when providing researchers with access to data, based on “safe people, safe projects, safe settings, safe data, safe outputs” [3].

‘Safe output’ guidelines are typically aimed at traditional statistical outputs such as regressions, graphs, and tables. Such outputs are human-readable and can therefore be manually assessed by TRE output checkers to ensure that they do not disclose any sensitive information. TREs have typically no experience dealing with outputs that are not amenable to manual validation [4], which risks weakening the “safe outputs” pillar unless new assistance can be provided to TRE output checkers.

Machine Learning (ML) is a subfield of AI in which algorithms are trained to perform tasks by exposing them to large quantities of relevant data. It is widely considered that ML systems will play an increasing role across society. It comes as no surprise then that there is an increased interest in training ML models on the data that is held within TREs.

A researcher training an ML model within a TRE is most likely to export the trained ML model, and deploy it into a useable pipeline. A trained ML model is typically not stored in a human-readable format; and most models are not amenable to manual checking – due to complexity. Determining how to categorise these models (in terms of AI systems), the levels of risk involved (including security risk), and the checks and safeguards needed (e.g. human and public oversight), are important initial considerations to make [5]. Stringent measures are required to ensure data privacy once the trained model is deployed outside the TREs.

TREs provide approved researchers with a single location to access datasets where the ML models can be trained on sensitive health data. Unlike TREs, Personalised Health Trains (PHTs) enable federated learning by allowing the training of ML models in multiple iterations at different secured sites [6]. Both these secure environments enable safe analysis, however, it is unclear if they are well-equipped to ensure that the trained model contains no sensitive information and is safe to deploy.

To understand better how this issue is being addressed in practice, in a recent survey, we interviewed 14 UK and 6 international TREs [7] to discover current processes within TREs for AI algorithms disclosure. Our findings showed that, across the board, TREs did not have mature processes, tools, or an understanding of disclosure control for AI algorithms, relying on a degree of manual checking that is likely not fit for purpose. The disclosure risks posed by trained models can be subtle, and just because an output checker cannot “see” something disclosive within the model, does not remotely suggest that it is safe. This demonstrates a worrying gap between how ML techniques are being developed and the governance and oversight of the TREs, resulting in risks to privacy, data protection and good governance of TREs.

This paper differs from a typical review article in its methodological pattern. It focuses on articulating and explaining AI in the context of TREs, raising the awareness of associated risks and potential technical and legal controls to a new audience of health data researchers, who aim at experimenting with various ML models within TREs and with TRE providers. This is a recent, but rapidly growing field of research with relatively few studies. To access these, we used a combination of keyword searches on a range of sources including arXiv, IEEE Explore and ACM using the terms *Trusted Research Environment, Safe Haven, AI, ML, Data Privacy, and Disclosure Control*. As TREs can be assumed not to be malicious actors we have excluded work on attacks on Federated Learning from our review. Section 2 (Background) discusses some popular ML models and the kind of information present in a saved ML model. We highlight, for a non-expert audience, the ways an ML model can disclose personal information regarding the data on which it is trained. Section 3 (Risks) assesses the risks of such disclosures, but now within the context of TREs. Finally, Section 4 (Discussion) summarises the areas where research is needed, to provide the research community and TREs with the knowledge and tools required to safely disclose trained ML models.

## 2 Background

### 2.1 ML in healthcare

The combination of algorithmic advances and the availability of large datasets has led to increased use of AI and particularly ML. This interest is particularly high in the medical and healthcare fields where the adoption of these approaches can potentially improve efficiency, reduce the burden on clinicians, and assist more predictive and preventative philosophy to care [8]. However, this use of AI must be tempered by due diligence of AI's strengths and limitations [9].

ML is a subset of AI, that encompasses many computational algorithms that automatically *learn* patterns from datasets [10]. It can be used to help humans better understand complex data, or make predictions on new, unseen data. Recent technological advances, coupled with the increased availability of large datasets have resulted in increased popularity.

Table 1 summarises how ML models can be split into various categories, based on the type of problem they are attempting to solve.

**Table 1:** Categories of ML models.

**Supervised Learning**	Algorithms are supplied with a corpus of training examples, each consisting of some input values associated with an output category or value (a *label*). For example, the data about a patient is labelled with whether a patient had cancer or not. The goal is for the algorithm to learn a mapping between the input and output that can subsequently be used for prediction. In our example, the input would be the data about a patient and the output would be a prediction of cancer. The output is typically either one of several distinct categories (classification), e.g., ‘case’ vs ‘controls’, or a real value (regression). This is the most common type of ML [11] and is used for predicting risks of future health events. It has been used with a wide range of healthcare and medical data [[12], [13], [14]].
**Unsupervised Learning**	Algorithms use unlabelled data to discover patterns in a dataset. The most common example of unsupervised learning is clustering [15] which aims to group data instances such that the instances sharing a cluster are similar to one another. Unsupervised learning techniques are often used for exploratory data analysis in healthcare and medical applications. For example [16], used k-means clustering for identifying several subtypes of Alzheimer's using Electronic Health Records (EHR). More recently unsupervised learning was used to learn appropriate features for COVID-19 diagnosis from CT medical imaging [17].
**Semi-supervised Learning**	Algorithms are applied to datasets in which both labelled and unlabelled examples co-exist, with typically far more unlabelled than labelled records. This scenario is common when the cost of labelling data is high which is often the case in the healthcare and medical domains [18]. Semi-supervised methods use patterns present in the unlabelled data to improve performance over models trained on the labelled data alone. These approaches can also be used iteratively, identifying at each iteration which of the unlabelled points it would be best to acquire the label of by, for example, querying a human expert (Active Learning) [19].
**Reinforcement Learning:**	Reinforcement learning (RL) algorithms learn by receiving feedback as they interact with their environment [20], rather than from a static collection of training data RL has had high profile success in learning game strategies, where rewards are received at the end of a (variable length) game [21]. Examples of RL in healthcare include medical imaging [22], diagnosis systems [23] and precision medicine [24].
**Self-supervised learning**	Algorithms (typically deep learning models) derive representations of data without requiring labels. Self-supervised learning (SSL) has emerged recently, especially in the computer vision field, and [25,26] proposed the use of SSL method for anomaly detection in medical images.

### 2.2 Common ML models

Within the broad categories defined in Table 1, ML models can be further grouped according to their purpose, algorithm type and how they learn [10]. Table 2 and Table 3 summarise the most common types of ML models categorised by the type of algorithms used, and how they handle data for supervised and unsupervised learning.

**Table 2:** Supervised ML model types.

Algorithm type	Definition	Models	Examples	Reference
Traditional regression/parametric based methods.	Typically, a single parameter needs to be learnt per input feature.	Linear Regression Logistic Regression Stepwise Regression Multivariate Adaptive Regression	Medical images landmark detection	[27]
Instance/non-parametric based classification methods.	Methods based on a ‘similarity’ measure between data instances. Predictions are made by summarising the outputs for training examples. Weights are increased for data that is more similar to new observation.	K-nearest Neighbor (KNN) Self-Organising Map (SOM) Support Vector Machines (SVM)	Decision-making in mental health systems using SOM	[28]
Tree-based Algorithms	Methods that make decisions based on traversing a tree. The value of features at each node is used to decide the direction of the decision. Typically, recursive search methods are used to successively grow trees, or select nodes.	Classification and Decision Tree Chi-squared Automatic Interaction Detection (CHAID) Conditional Decision Trees	Analysis of adverse drug reaction using CHAID	[29]
Bayesian Algorithms	Probabilistic algorithms based upon Bayesian statistical principles. These methods operate by using data to update prior probabilities (e.g. class membership) to posterior probabilities.	Naïve Bayes Bayesian Belief Network (BBN) Gaussian Naïve Networks	Modelling fetal mortality	[30]
Neural Networks and Deep learning	Loosely inspired by biological neural networks, they consist of non-linear ‘neurons’ organised into layers. Typically an input layer, is connected to one or more hidden layers, followed by an output layer. Deep neural networks have many hidden layers and provide state-of-the-art performance for complex data such as images, text and audio.	Convolution Neural Networks (CNN), Recurrent Neural Networks (RNNs) - for example, Long Short-Term Memory networks (LSTM), Auto-Encoders, Deep Belief Network (DBN)	CNN for the classification of skin cancer LSTM for predictive medicine from EHR. Genomic data imputation using autoencoders	[31] [32]
Natural Language Processing (NLP)	NLP methods aim to process and understand language in the form of text or speech and are widely used in the medical field. Classical statistical and rule-based approach to NLP, have recently been augmented by ML, especially the use of transformers	Computational linguistics Statistical and ML models Deep learning and transformers	Computational linguistics to extract cancer phenotypes Use of BERT in EHR for disease prediction	[33] [34]

**Table 3:** Unsupervised ML categories.

Algorithm type	Definition	Models	Examples	Reference
Non-parametric Clustering Algorithms	Group (partition) the data observations according to similarity between them.	Hierarchical Clustering Kernelized K-means	Alzheimer's structural imaging phenotype Detection using hierarchical clustering. Diabetes diagnosis using K-means clustering	[35]
Parametric Clustering Algorithms	Define groups of data using parametric model. Data is assigned to cluster using the largest prior probability	K-means K-medians Statistical Mixture models Gaussian Mixture Models	Diabetes diagnosis using K-means clustering	[36]
Admixture models	Whereas clustering assumes that each data point belongs to a single cluster, admixture approaches allow each data point to take contributions from multiple clusters (aka topics). Popular in natural language processing.	Latent Dirichlet Allocation	Modelling tabular healthcare data	[37]
Dimensionality reduction	Algorithms that compress high-dimensional data into a low-dimensional representation. The aim is to preserve all useful structures of the data in an unsupervised way. The resulting low-dimensional representations are used for data exploration and visualisation. They can also be used as features for other ML models	Principal Component Analysis (PCA) Multi-dimensional Scaling (MDS) T-Stochastic Neighbor Embedding (TSNE)	Brain tumour segmentation	[38]
Neural Networks and Deep Learning	These methods have been defined in Table 1. They can have both supervised or unsupervised learning			

### 2.3 ML model development

**Fig. 1:**
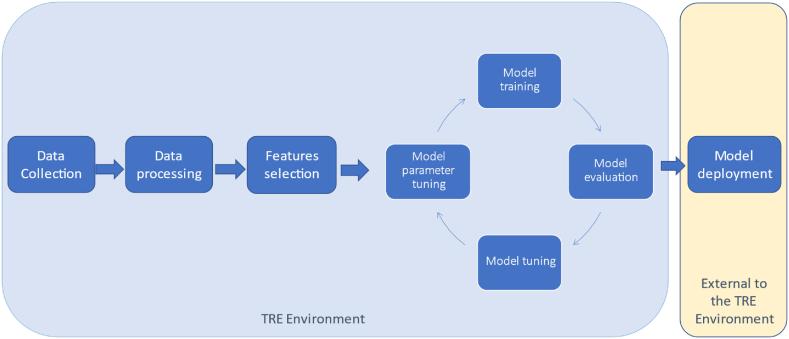
ML model development pipeline.

Fig. 1 shows the steps in the development of a ML model from data collection through to final deployment. For a model trained within a TRE all steps apart from initial collection and deployment would occur within the TRE, and deployment would necessitate removing of the model from the TRE.

Trained ML models are defined by values for parameters that are optimised during learning. This process is governed by hyper-parameters (e.g. learning rates for neural networks) that are usually set based on expert or domain knowledge or through trial and error. Thus, finding the model with the best-estimated performance for the desired task is often an iterative process.

A key step in model development, particularly for supervised models, is validation of the model's performance. Performance on previously unseen test data should be used to estimate how well the model will generalise to unseen data, and hence evaluate its performance on deployment. The validation process also highlights when models have simply memorised the training data rather than learning useful structure, an issue known as overfitting [39].

### 2.4 Outputs of a ML training process

A researcher wishing to deploy a trained ML model outside TRE may export two types of output: performance analysis, and the trained model itself. The former would fall within the remit of traditional output checking. However, the latter would not, and we will now explore what kind of information would be held within a trained model file.

#### 2.4.1 Architecture

This is most relevant in neural network (and therefore deep learning) models where the architecture defines the number of nodes in each layer, how they are connected, and the activation functions used in each layer. In our definitions we consider architecture to be defined by hyper-parameters, and set *before* training so, e.g., the definitions of the nodes (features and thresholds) in a decision tree are *parameters* rather than *architecture*.

#### 2.4.2 Parameters

A model's parameters are variables within a model that are optimised during training. For example, in logistic regression, these are the weights that each input variable is multiplied by in the decision function. For a neural network, these are the weights on the connections between neurons.

#### 2.4.3 Configuration

Many models save the details of any additional parameters that were set by the user for training, possibly including some information regarding the dataset that was used for training, for example variable names.

#### 2.4.4 Optimiser and its state

To facilitate ongoing/restarting training, some models (especially neural networks) will by default store the state of the optimisation process at the time it is stopped. This can include which optimiser was used, associated parameters (e.g., learning rate), how many iterations the optimiser ran for, and why it terminated (convergence, the maximum number of iterations was reached, etc). Saving the optimiser state can be useful if further training is desired.

#### 2.4.5 Training data

Some algorithms require storing some or all training examples to make predictions (e.g., KNN and SVMs). There are various stages in the ML development/deployment cycle when the models become vulnerable to attack. For example, the training phase can be compromised if training sets are poisoned, and deployed systems can be subject to adversarial attacks. Although these threats are important, we are interested in specific risks around disclosure of personal data once a model is outside a TRE, which arise through membership inference (MEI) and model inversion attacks (MOI) [[40], [41],]. For a comprehensive review of all vulnerabilities of trained ML models, the reader is referred to [42].

Below, we describe MEI and MOI attacks in more detail. In both cases, we assume the attack is undertaken by a malicious actor (the attacker) outside the TRE with access to the trained model, either directly, or by sending a query with data through an interface and recording its predictions. Note that such attacks require some effort on the part of the attacker (computational or otherwise). This is different from other breaches where a record is made available in ‘raw form’ (the classic ‘laptop on a train’ scenario and more modern variants) – which should be covered by standard data management policies.

#### 2.4.6 MEI attacks

MEI attack was introduced by [43], where an attacker having access to some particular data instances attempts to determine if they were part of training data.

MEI attacks leverage the observation that models will often make more confident predictions on data that they were exposed to in training than unseen data. Therefore, high predictive confidence can infer the likelihood of a data point belonging to a training set. Overconfidence in training examples is associated with model overfitting [44] resulting due to poor design of model architecture or fewer training examples. This has been demonstrated by [45], who also experimented with MEI attacks on federated learning systems where a model is trained on data from various locations simultaneously. To perform MEI attacks, an attacker would train an attack model with the predictive probabilities produced by the model being attacked (the target model) for data that was included and not included for training. As the attacker cannot access training data, they typically train their own versions of the target model (known as shadow models). Although the attacks are still possible based on shadow models that neither share architecture nor training data with the target model [46], the more information attacker have about the original data and the target model, the higher the success rate.

MEI attacks can be performed on NLP applications such as in [47], where Carlini et al. demonstrated that even without overfitting, large language models such as GPT-2 can still memorise sensitive data. Similarly, Vakili et al. [48] investigated the privacy preservation of language models such as BERT in clinical data.

#### 2.4.7 MOI attacks

MOI attacks, (also known as attribute inference attacks) attempt to infer aspects of the input training data. These attacks can be particularly dangerous for private and confidential data [49]. For example, an attacker might attempt to infer some particularly sensitive model inputs for one or more individuals based on other input values and the model's predicted output, both of which may be straightforward to obtain e.g., through social media or from news stories (for famous individuals).

Fredrikson [50] introduced the concept of MOI by showing how a model that predicted drug dosage could be inverted to leak sensitive data about individuals in training data. Recent examples include Nigesh et al. [51] who analysed deep learning models trained on 3D brain image segmentation tasks. Knowledge of the model type and architecture can assist MOI attackers in reproducing the parameters or functionality of a model. For example, knowing the model is a logistic regression classifier, the attacker knows the structure and, by presenting sufficiently diverse inputs to the model and recording the outputs, can construct a series of equations from which the regression weights can be reverse-engineered. If attackers are just interested in mimicking the functionality, presenting many input examples, and recording the outputs would train a completely new model.

## 3 Risks

### 3.1 Risks of exporting training ML models from TREs

Based on the background presented in the previous sections, we believe that there are significant risks that must be addressed before TREs can safely allow export of trained ML models. In this section, we summarise these challenges, categorised under users, data and ML models.

#### 3.1.1 Users

Threats around trained ML model disclosure can emerge from different users and actors, of whom we identify four (Table 4).

**Table 4:** Different users and actors who are potential threats.

**Non-malicious researchers**	Many TRE users will likely have little awareness of threats introduced by trained ML models. For example, it is easy to imagine a researcher training a SVM, unaware that the saved SVM has to include a copy of at least part of the training dataset for it to operate - a considerable disclosure risk. Similarly, although most ML developers would understand the concept of over-fitting, they are unlikely to realise its link with vulnerability to MEI attacks.
**Malicious researchers**	These are users who deliberately hide data inside disclosed models and outputs. They directly cause a data breach, by dissimulating data inside other outputs. To a certain extent, malevolent behaviour is guarded against, through the existing TRE safeguarding procedures [1]. However, these guidelines were designed for aggregated results (tables, plots, or summary statistics), in which the possibility of hiding large quantities of data was insignificant. This is different when disclosing ML models, where files being disclosed could be large and not necessarily human-readable.
**External attackers**	The third actor is an external attacker who has access to trained models after they have been disclosed from the TRE, through either model deployment (e.g., via an accessible application programming interface; API) or model sharing. Attacks can be carried out in various forms as described above.
**TRE output checkers**	The final actors are the TRE output checkers themselves. Given the complexity of ML models, it is highly likely that the majority have little or no familiarity with those types of outputs and are thus being asked to check things that they do not understand, to assess risks that they also do not understand.

#### 3.1.2 Data

The application of ML in healthcare represents more challenges when disclosing models from TREs. Electronic Health Records can be highly heterogenous and includes both structured and unstructured data such as medical images, and large genomic databases. Within TREs, data is safeguarded and follows strict protocols such as de-identification [52], anonymisation, and pseudonymisation [53]. De-identification consists of removing any personal direct identifiers. Anonymisation aims at removing any personal identifiers from data resulting in individuals being completely unidentifiable.

Pseudonymisation on the other hand represents the process of replacing certain identifiers, where data can no longer be linked to personal identifiers without recourse to further processing [54].

Depending on the data types being considered, TRE operators need to adopt appropriate methods for removing personal data. Whereas in traditional analysis, an output checker could see the output and assess if the process followed by the researcher was exposing sensitive data, this is substantially more challenging with a trained ML model.

#### 3.1.3 ML models

Privacy-breaching the model, where they cannot access the model file itself but only query the model. Attackers can therefore *use* the model (present inputs and be provided with outputs) but cannot observe its inner workings. In some cases, an attacker might get access to the model file (a *white box* attack) and therefore to model parameters and architecture. White box attackers can both use and inspect the model [39]. In general, white box access confers greater risk. However, black box attacks can also represent a significant risk but can require more effort to be successful.

The challenge of disclosing ML models varies between different models and training regimes. Some models are more prone to attacks than others, although model configuration (architecture and setting of model hyper-parameters) also plays a significant role. Disclosing an SVM or a tree-based algorithm may be riskier than disclosing more complex models [55]. More work is required to better understand the risk of a wide range of models and configurations.

### 3.2 Legal and regulatory considerations

The personal data used in TREs, which may be exported in a trained ML model, will be governed by data protection law if it exists in a particular jurisdiction. Internationally, the European Union's General Data Protection Regulation (GDPR) is the most prominent data protection framework, and the UK implemented it into domestic law in the Data Protection Act 2018. Given our focus on UK-based TREs, we proceed with some key points about its application.

The key challenge is responsibility for a possible data breach resulting from the export of a ML model. Understanding that the disclosure of a trained ML model, which works differently from the disclosure of standard statistics (e.g. graphs and tables etc.), is important. The security of processing is a responsibility attributed to the controller and the processor, as outlined in Article 32 of the GDPR. The controller and processor must take “state of the art”, technical and organisational measures into account. These include “appropriate” measures commensurate with the risks, in terms of (a) pseudonymisation and encryption, (b) ongoing resilient systems and services, (c) ability to restore personal data (PD), and (d) regular testing for effectiveness of the security of the processing [56]. For a trained ML model, the chain of responsibility may be unclear where the initial data controller and TRE processor are no longer involved when the ML model has been transferred out of the TRE, and if the responsibility for any potential future personal data breach has not been identified nor allocated correctly in the terms of an agreement that a TRE operator has made with a TRE user. Assessing the level of risk as to whether the trained ML model and/or its output are inextricably linked to potentially disclosive personal data [57] it was trained on will determine how the law categorises the model. We consider there are 3 categories:

1. A trained ML model can be considered to only contain anonymous data and therefore GDPR does not apply [58].
2. A trained ML model is considered to potentially contain pseudonymised personal data, therefore requiring specific technical and organisational measures to ensure the processing is GDPR compliant. In particular, whether appropriate data security measures have been adopted to reflect the risk of a data breach.
3. A trained ML model is considered to carry more risk of including disclosive personal data (pseudonymised) therefore requiring extra layers of protection in terms of the transfer of responsibility, obligations and rights to a new data controller/processor, with prior written authorisation or consent from the controller, or indeed the retention of responsibility, obligations and rights by the original data controller in the contract [59].

### 3.3 Risk analysis in disclosing models from TREs

**Fig. 2:**
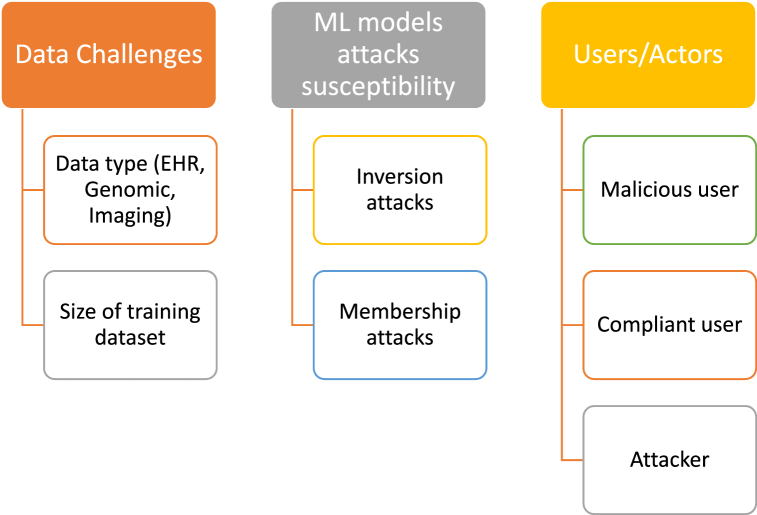
Factors that impact the disclosure of trained ML models.•***Data types***: The heterogeneity of healthcare and medical data contribute to the different levels of risks associated with disclosing models from TREs. In fact, some types of data can pose greater risks than others. Clinical text and clinical images have a different level of risk in the re-identification of personal and private data, for example, an X-ray of a knee is less disclosive than clinical phenotype for rare diseases.•***ML types***: In addition to different data types, using different ML models can alter the risk level. Some models pose more risks than others as demonstrated in [47], where large language models may remember data even if overfitting is reduced.•***Actor types:*** Different actors will have different effects on preserving privacy in ML models. Outsider attackers will aim at reconstructing part or all of the training data. This would negatively affect the risk of disclosure of models. Similarly, a user that follows processes to create privacy-preserving models would pose a lower risk than a less knowledgeable user.

Preserving data privacy when disclosing ML models is challenging. For a model to be effective, it must retain some aspects of the data on which it was trained [41]. Disclosing ML models from safe environments, therefore, carries risks that must be accounted for, analysed and mitigated. We have identified various factors that may affect the level of risks such as data types, ML type or attack type. Fig. 2 summarises the above-mentioned factors.

In addition, the combination of different factors contributes to the level of risk. For example, disclosing an SVM model trained on brain MRI data could represent a higher risk of re-identification than a deep learning model using genomic data as medical images can contain hidden biometrics [60], and pose a higher risk to privacy if reconstructed via a MEI attack [61].

To assess the risk of disclosure, TREs need to consider the severity of the different factors (alone or in combination) and the impact of a data breach, which would primarily concern the re-identification of individuals and subsequent loss of trust in TREs. This can be achieved by conducting a thorough risk analysis before the disclosure of ML models.

## 4 Discussion

In this paper, we have drawn attention to a new problem facing TREs: the output of trained ML models. Our previous research [4] has demonstrated that TREs are ill-equipped for this task, although it is likely that requests to remove trained models from TREs will become increasingly common. As well as describing TREs, we have provided a brief introduction to ML, and the privacy risks that a trained ML model can present.

The challenges and risks described in this paper create new opportunities for interdisciplinary research. We have recently carried out preliminary research in this area developing a set of implementable recommendations for the disclosure of trained models from TREs [62]. We investigated not only disclosure controls but also model query controls as methods which could be adopted. Others have also developed methods for auditing the risk of ML models [63], but not in the context of TREs. However, there remains much need for research in this area:

• ***Data privacy research:*** Various opportunities exist in providing more secure processes at the data level. This could include encryption of the data and enabling the application of ML algorithms directly on encrypted data e.g. homomorphic encryption. Adding digital watermarks enables data tracking that could help detect privacy leaks [64]. ML models could also be trained on synthetic data. However, this method can only be applied to very limited types of data and applications applying synthetic data in the medical field can lead to algorithm bias [65]. TREs can also provide guidelines for researchers and users to ensure data privacy throughout the whole ML models life cycle; where users need to ensure data privacy during training, input privacy, output privacy and models privacy.
• ***ML Model evaluation*:** The community needs large-scale evaluation studies to attempt to uncover the vulnerabilities of common ML models. Although this area has progressed, much more experimental work is needed to assess the wide range of models that researchers may wish to use in TREs. New methods of training models that aim to improve privacy (e.g., differentially private model training [[49], [67]], and federated learning [66]) must also be evaluated. The results of these analyses must be made accessible to TREs controllers.
• ***Automatic ML model privacy assessment:*** Quantifying risks from ML models is impossible using human controls only. Providing TREs and researchers with tools that help assess risk factors for models can help quantify, manage and mitigate risks in the disclosure of such models, e.g., the tools provided in [68]. Various ML attack metrics tools have been proposed in the literature such as those in [12].
• **AI responsibility and accountability:** Exploring areas such as accountability, and explainability, from both, technical and legal point of view. In addition to explainable AI, the legal community will need to be able to prove who and/or what was responsible for a data breach (causality) [69]. If we consider that the GDPR allocates responsibility to a person (controller/processor/sub-processor) when determining who is responsible for a breach in testing the effectiveness of the de-identification of personal data, it is clear that current UK GDPR law regulates for responsibility to be attributed to a person [70].
• ***Model-based mitigation strategies and guidelines:*** Develop methodologies for mitigating risks linked to model disclosure such as restricting types of models that can be used. Some model types such as KNN and SVM represent a higher risk and need tighter control.
• ***Effective manual disclosure procedures***: This could include model/code inspections before disclosure. This requires experts within the disclosure teams, and the technical infrastructure to generate and store code snapshots for inspection. Ideally, the disclosure team would also use the model, independently evaluating model performance on both the data that was provided to the researcher and a subset of unseen data. For example, 10% of the examples could be withheld from the researcher for use by the disclosure team.

## 5 Summary table

**Table:** 

What was already known on the topic	• AI/ML is increasingly being used in healthcare provision and there is a demand to train new algorithms on health data. • Trained AI/ML models can potentially encode personal data and be attacked. • TREs do not have mature processes, tools, or an understanding of disclosure control for AI/ML algorithms, relying on a degree of manual checking that is likely not fit for purpose.
What this study added to our knowledge	• A new understanding of the risks to disclosing personal data from trained ML models within the new context of TREs. • Research is urgently needed to form the basis of new output checking procedures for TREs.

## Author contribution statement

All authors listed have significantly contributed to the development and the writing of this article.

## Funding statement

Professor Emily Jefferson was supported by 10.13039/501100000266Engineering and Physical Sciences Research Council [MR/S010351/1]; 10.13039/501100000265Medical Research Council [MR/S010351/1].

## Data availability statement

No data was used for the research described in the article.

## Additional Funding Information

This project was in part supported by MRC and 10.13039/501100000266EPSRC program grant: Interdisci**Pl**Inary **C**ollaboration for efficient and effective **U**se of clinical images in big data health care **RES**earch: PICTURES [grant number MR/S010351/1].

This work was in part supported by Health Data Research UK (HDR UK: 636000/RA4624) which receives its funding from HDR UK Ltd (HDR-5012) funded by the 10.13039/501100000265UK Medical Research Council, 10.13039/501100000266Engineering and Physical Sciences Research Council, 10.13039/501100000269Economic and Social Research Council, 10.13039/501100000276Department of Health and Social Care (England), 10.13039/100014589Chief Scientist Office of the Scottish Government Health and Social Care Directorates, 10.13039/501100010756Health and Social Care Research and Development Division (Welsh Government), 10.13039/501100001626Public Health Agency (Northern Ireland), 10.13039/501100000274British Heart Foundation (BHF) and the 10.13039/100010269Wellcome Trust.

This work was in part supported by the Industrial Centre for AI Research in digital Diagnostics (iCAIRD) which is funded by Innovate UK on behalf of 10.13039/100014013UK Research and Innovation (UKRI) [project number: 104690].

This work was funded by 10.13039/100014013UK Research and Innovation Grant Number MC_PC_21033 as part of Phase 1 of the DARE UK (Data and Analytics Research Environments UK) programme, delivered in partnership with HDR UK and ADRUK. The specific project was Guidelines and Resources for AI Model Access from TrusTEd Research environments (GRAIMatter).

## Declaration of competing interest

The authors whose names are listed immediately below certify that they have NO affiliations with or involvement in any organization or entity with any financial interest (such as honoraria; educational grants; participation in speakers’ bureaus; membership, employment, consultancies, stock ownership, or other equity interest; and expert testimony or patent-licensing arrangements), or non-financial interest (such as personal or professional relationships, affiliations, knowledge or beliefs) in the subject matter or materials discussed in this manuscript.

**Dr Esma Mansouri-Benssassi,** Division of Population Health and Genomics, School of Medicine, University of Dundee, Dundee, Scotland.

**Dr Simon Rogers,** Artificial Intelligence Centre of Excellence, NHS National Services Scotland, Glasgow, Scotland.

**Dr Smarti Reel**, Division of Population Health and Genomics, School of Medicine, University of Dundee, Dundee, Scotland.

**Maeve Malone**, School of Humanities Social Sciences and Law, University of Dundee, Scotland.

**Prof Jim Smith**, School of Computer Science and Creative Technologies, University of West England, Bristol, England.

**Prof Felix Ritchie,** FBL - Accounting, Economics and Finance, University of West England, Bristol, England.

**Prof Emily Jefferson**, Division of Population Health and Genomics, School of Medicine, University of Dundee & HDR UK.
